# Visual Prompting based Incremental Learning for Semantic Segmentation of Multiplex Immuno-Flourescence Microscopy Imagery

**DOI:** 10.21203/rs.3.rs-3783494/v1

**Published:** 2023-12-25

**Authors:** Ryan Faulkenberry, Saurabh Prasad, Dragan Maric, Badrinath Roysam

**Affiliations:** 1 Department of Electrical Engineering, University of Houston, 4226 Martin Luther King Boulevard, Houston, 77204, Texas, United States.; 2 Flow and Imaging Cytometry Core Facility, National Institute of Health, Bethesda, 20814, Maryland, United States.

**Keywords:** Image segmentation, transformers, incremental learning

## Abstract

Deep learning approaches are state-of-the-art for semantic segmentation of medical images, but unlike many deep learning applications, medical segmentation is characterized by small amounts of annotated training data. Thus, while mainstream deep learning approaches focus on performance in domains with large training sets, researchers in the medical imaging field must apply new methods in creative ways to meet the more constrained requirements of medical datasets. We propose a framework for incrementally fine-tuning a multi-class segmentation of a high-resolution multiplex (multi-channel) immuno-flourescence image of a rat brain section, using a minimal amount of labelling from a human expert. Our framework begins with a modified Swin-UNet architecture that treats each biomarker in the multiplex image separately and learns an initial “global” segmentation (*pre-training*). This is followed by incremental learning and refinement of each class using a very limited amount of additional labeled data provided by a human expert for each region and its surroundings. This incremental learning utilizes the multi-class weights as an initialization and uses the additional labels to steer the network and optimize it for each region in the image. In this way, an expert can identify errors in the multi-class segmentation and rapidly correct them by supplying the model with additional annotations hand-picked from the region. In addition to increasing the speed of annotation and reducing the amount of labelling, we show that our proposed method outperforms a traditional multi-class segmentation by a large margin.

## 1 Introduction

Accurate image semantic segmentation is the foundation of many automated clinical diagnosis tools, and improvements in semantic segmentation have immediate downstream benefits to computer-aided diagnosis systems that rely on robust segmentation of organs or tissue.

In recent years, deep learning based semantic segmentation has shown promise for medical image segmentation tasks. The CNN-based UNet [1], for example, is the *de facto* standard for medical segmentation [2]. By using an encoder-decoder architecture with skip connections to combine hierarchical features, UNet has been shown to provide good semantic segmentation performance for many tasks. Due to it’s success and widespread adoption, many variants have been developed for different segmentation domains [3–5], such as Attention UNet [6], DenseUNet [7], and UNet++ [8]. Attention UNet filters features passed through skip connections at each scale with a mechanism called an attention gate. The function of the attention gate in the context of Attention Unet is to suppress feature activations in irrelevant regions of the image, improving segmentation accuracy. Attention coefficients at each gate select only the feature responses that preserve activations relevant to the specific task. Existing features are multiplied with these coefficients element-wise to produce the skip connection output. DenseUNet uses dense blocks (a densely-connected set of convolutions) to extract features rather than single convolutions, with the aim of bringing the benefits of DenseNets to the segmentation of microscopy images. The addition of these dense blocks has benefits such as reducing the number of trainable parameters without reducing network depth, and inherent regularization, which is particularly useful in the usual medical imaging situations where few annotated training cases are available. UNet++ adds nested and dense connections between the encoder and decoder at each scale, in addition to the skip connections found in a plain UNet. By convolving feature maps from the encoder prior to fusion with the decoder, the two feature maps are made more semantically similar, improving segmentation.

More recently, inspired by the UNet and the recent success of vision transformers for computer vision tasks, Swin-Unet [9] was developed as a transformer-based variant of the UNet. Originally used in natural language translation [10], transformers pass an input sequence to an encoder, where features are extracted with alternating self-attention and fully-connected layers, with residual connections bypassing each layer. This feature sequence is then decoded in a similar fashion with groups of self-attention, fully-connected layers, and residual connections to form an output representation. By using positionally embedded patches of an image as the input sequence and adding a classifier, the language transformer has been adapted to successfully classifying images. Where a standard UNet uses convolutions at different scales for hierarchical feature extraction, Swin-Unet uses Swin Transformers [11] at different scales to accomplish the same. Because transformers and self-attention overcome the inherent locality of Convolutional Neural Networks (CNNs), Swin-Unet has been shown to outperform the standard convolutional based UNet architecture in publicly-available semantic segmentation benchmarks [9], as well as in private datasets [12, 13], making it the state-of-the-art in medical semantic segmentation.

Brain segmentation is most commonly performed on magnetic resonance (MR) and computational tomographic (CT) images due to the relative ease of data collection [14–17]. A different method, Immuno-fluorescence (IF) [18], is a nucleus-staining technique that allows visualization of many cells and synapses within a tissue segment. IF microscopy has been paired with semantic segmentation algorithms to better understand the underlying tissue structure [19]. Today, this work is dominated by CNN-based methods that have been adapted to microscopy images [20, 21]. Among these is Cellpose [22], a general segmentation framework designed to segment many different types of cells. Cellpose uses a variant of the watershed algorithm [23], a predeep-learning routine for cellular segmentation, to preprocess an input image into a topological map based on a human annotator. A custom UNet then classifies the gradients of the map to segment the image. Though competitive at instance segmentation, this method is not optimal for semantic segmentation, and may not be suitable for multi-channel data.

By leveraging a multitude of biomarkers when acquiring IF images, highly multiplexed IF microscopy [24] has the potential to provide a rich multi-channel representation of the tissue for accurate identification of all relevant cell phenotypes, which constitute the tissue and exhibit specific cell patterning distributions that define unique anatomical regions that make up an organ structure (e.g. within the rat brain), which can be used in downstream segmentation models.

Despite the promising developments in semantic segmentation algorithms, there are some pitfalls to consider when deploying them for biomedical imaging tasks (such as multi-channel IF microscopy). These deep learning models often require a large amount of labeled data (even more so for vision transformers that do not have inductive bias and need a lot of data to learn representations via self-attention). Additionally, recent developments such as the traditional UNet as well as the recent Swin-UNet were originally designed only for color and grayscale images and do not intrinsically leverage the rich multi-channel information provided by highly multiplexed IF microscopy imagery. Finally, traditional semantic segmentation frameworks consider the segmentation of the entire image as a single “global” task. This does not take into consideration the observation that analyzing “local” anatomical structures within the context of only surrounding anatomical regions can enhance the discrimination potential of segmenting out regions of interest.

In this work, we propose a multi-channel Swin-Unet (a Swin-Unet that has been adapted to extract information from highly multiplexed immuno-flourescence imagery) as well as a framework to incrementally fine-tune a multi-class segmentation network with a domain expert in the loop. The domain expert can help *steer* the fine-tuning of the network by providing a few representative patches (“clicks”) on the image per region. We show that exceptional segmentation can be produced with only a single training dataset and minimal additional labelling from the image where segmentation is desired, overcoming inherent limitations of state-of-the-art semantic segmentation models.

This paper is organized as follows: Section II describes related works in the area of medical image segmentation. Section III presents both our multi-channel Swin-Unet and our proposed framework of incremental fine-tuning a global semantic segmentation network. In Section IV, we discuss the experimental setup and results of applying this approach on an IF-stained rat brain imagery. Finally, we provide concluding remarks and possibilities for future work in Section V.

## 2 Related Work

Medical image segmentation is unique from other image processing problems in that most supervised learning paradigms require a large quantity of labeled data - something that is difficult to come by and often prohibitively expensive to attain. Thus, recent approaches to the medical segmentation problem have focused on making robust predictions from small datasets. The CNN-based U-Net has been applied with great success to many sparsely labeled biomedical datasets, and has since spawned a number of variants, owing to both it’s wide adoption in the biomedical research community and the diversity of medical imaging applications [2, 25, 26].

UNet is composed of an encoder, decoder, and skip connections that share information from the former to the latter after each downsampling operation. The encoder follows the structure of a typical CNN, composed of a collection of repeated 3×3 convolutions and max pool downsampling operations. The decoder has the same structure, but up-convolutions are used to upsample the data whereas max pooling was used for downsampling. Skip connections pass shallow, non-downsampled features from the encoder to the decoder, where they are concatenated with deep features which have passed through the entire model. The combination of upsampling and skip connections allows for unprecedented localization capability for a CNN model, even when applied to a small dataset.

Recently, transformer- and attention-based approaches to image classification and segmentation have gained popularity [27–29], outperforming CNN-based models at many tasks [30, 31]. The first transformer was designed for language translation, and eschewed convolutions and recurrence entirely in favor of an exclusively self-attention-based architecture [10]. Self-attention is competitive due to it’s ability to efficiently model long-term dependencies, which CNNs and recurrent networks inherently struggle with.

Dosovitskiy et. al. [32] developed the first competitive transformer-based vision model, Vision Transformer (ViT), and designed it to be as similar as possible to the preceding Natural Language Processing (NLP) Transformer architecture. Whereas a sequence of words forms the input to the NLP Transformer, a ViT input is formed by partitioning an image into non-overlapping patches, and feeding them to the transformer sequentially. Thus, in terms of the preceding NLP Transformer, each patch of the image is analogous to a word in a sentence.

Because sentences tend to be less informationally dense than images, and due to the quadratic time complexity of self-attention, ViT is not practical for use on large or high-resolution images. Out of the need for scaling came the Shifted-Window (Swin) Transformer. Instead of computing self-attention across the entire set of patches, The Swin Transformer partitions the set of patches into groups of non-overlapping windows, and computes attention for each window. To account for the lack of attention at the edges of the windows, a second transformer is applied with shifted windows that model connections between the first transformer’s windows, eliminating any “blind spots”. Thus, Swin Transformers always come in pairs. The shifted window method makes self-attention linear with respect to input size rather than quadratic, making the Swin Transformer feasible for processing large images.

The transformers described to this point are standalone image classifiers, but have been applied competitively to segmentation problems by inclusion of another module [33–35]. Swin-Unet, for example, gives competitive semantic segmentation results when used as an encoder for the CNN-based UperNet [36]. However, the semantic segmentation challenge of medical imaging is uniquely defined by a lack of training data, and the models described so far are not designed to account for this. Many models build on the existing CNN-based UNet architecture by incorporating transformers in creative ways. TransUnet, for example, uses ViT as an encoder within a UNet-like CNN architecture [37]. TransFuse [38] runs a CNN and transformer encoder in parallel and fuses the results. One very well-performing medical segmentation model is the Swin-Unet [9]. Swin-Unet is unique from the other mentioned architectures in that it is completely self-attention based, convolutions are used only for patch partitioning and embedding (rather than feature learning), and recurrence is eschewed entirely. The Swin-Unet achieves state-of-the-art performance by using a series of Swin Transformers in conjunction with a transformer-specific downsampling operation to encode deep representations. Encoded features are then upsampled and combined with features from the encoder via skip connections, yielding a segmentation in a very similar fashion to the CNN-based UNet.

## 3 Proposed Work

In this section we introduce our incremental learning approach to semantic segmentation of multi-channel images. We begin by describing a custom Swin-Unet model and it’s advantages over an off-the-shelf model in the context of our multiplex immuno-flourescence microscopy imagery. We then describe our proposed method to incrementally fine-tune the “global” model for image sub-regions, aided by a human domain expert. Finally, we provide a strided inference method to retain smoothness between image patches.

### 3.1 Multi-Channel Swin-Unet

**Fig. 1:**
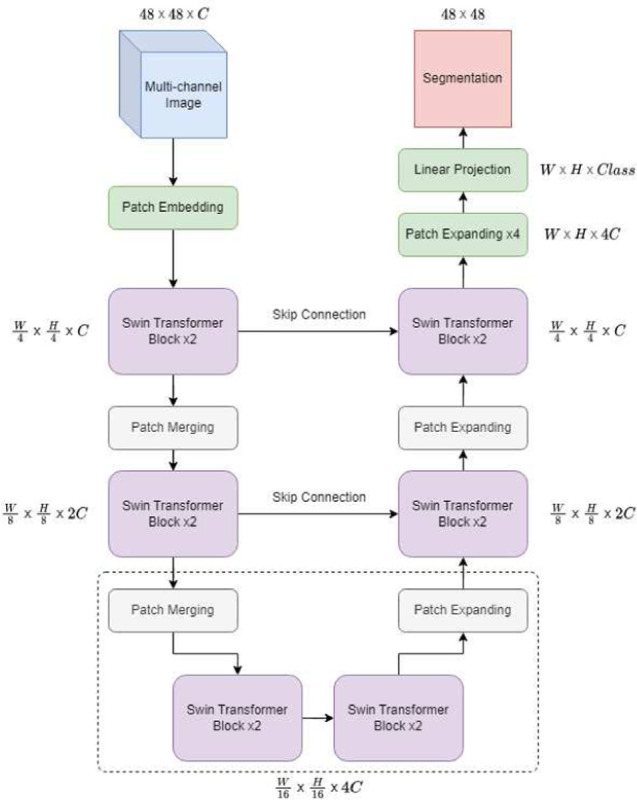
The multi-Channel Swin-Unet architecture

Our custom Swin-Unet is shown in Fig. 1. We opt to use a 48 × 48 input size rather than the standard 224 × 224 for our model. This window size provides the spatial context needed to capture the region specific phenomena such as cells and neurons and their immediate neighborhood.

#### Patch Embedding:

Both the standard Swin-Unet and our version begin with partitioning of the image into patches and embedding them. To adapt our model to multi-channel data, patch partitioning and embedding is performed by a single, separable 2D convolution layer, whereas the original Swin-Unet uses a non-separable convolution, since it’s intended domain is grayscale and RGB images. For a patch size of 4 × 4× *n*, we separably convolve the 48 × 48 × *n* image with a window size and stride of 4 (the patch size). We set 12 filters per biomarker channel for the separable convolution, for a latent output depth of *C* = 96, following the original Swin-Unet model. The output of this operation is a non-overlapping set of 4 × 4 × *n* patches projected to *C* feature channels. The patches in the sequence are vectorized and the sequence is passed to the encoder, which is composed of repeated Swin Transformer and patch merging blocks.

#### Swin Transformers:

Swin Transformer pairs split the input patches into windows and compute multi-head self-attention (MSA) within each window. The self-attention operation allows the model to weigh the importance of different patches in the context of all other patches in the window, and dynamically adjust their influence on the output.

#### Multi-Head Self-Attention:

The transformer computes MSA from three equivalent copies of the input sequence x. By convention, these copies are known as the query, key, and value vectors, denoted Q, K, and V, where Q, K, V, $x\in R^{n\times C}$. n is the number of patches in the input sequence and C is the latent channel depth of each patch. For multi-head attention, each of Q, K, and V are partitioned channel-wise into h sub-vectors, such that each sub-vector $Q_{i},K_{i},V_{i},i\in 1,\ldots ,h$ is of the following shape: $Q_{i},K_{i},V_{i}\in R^{n\times \frac{C}{h}}$. For each triplet $\left(Q_{i},K_{i},V_{i}\right)$, the vectors therein are each linearly projected back to $R^{n\times C}$ and an attention head is assigned to the triplet.

Having multiple heads work on different segments of the feature space in parallel enables the model to efficiently attend to multiple representations of the input sequence. For example, one attention head may model relationships between adjacent patches, while another models relationships between distant patches. For each head, self-attention itself is computed as follows:

(1) $$Attention({\tilde{Q}}_{i},{\tilde{K}}_{i},{\tilde{V}}_{i})=softmax(\frac{{\tilde{Q}}_{i}{\tilde{K}}_{i}^{T}}{\sqrt{C}}{\tilde{V}}_{i}),$$

where ${\tilde{Q}}_{i}=QW_{i}^{Q}$, and $W_{i}^{Q}$ is the parameter matrix of the linear projection of $Q_{i}$. Self-attention models the relationship between features of each patch to features of other patches. The output matrix gives the strength of the relationship between patches. Finally, the outputs of the attention heads are concatenated and the result is linearly projected back to the input dimension.

#### Windowing:

**Fig. 2:**
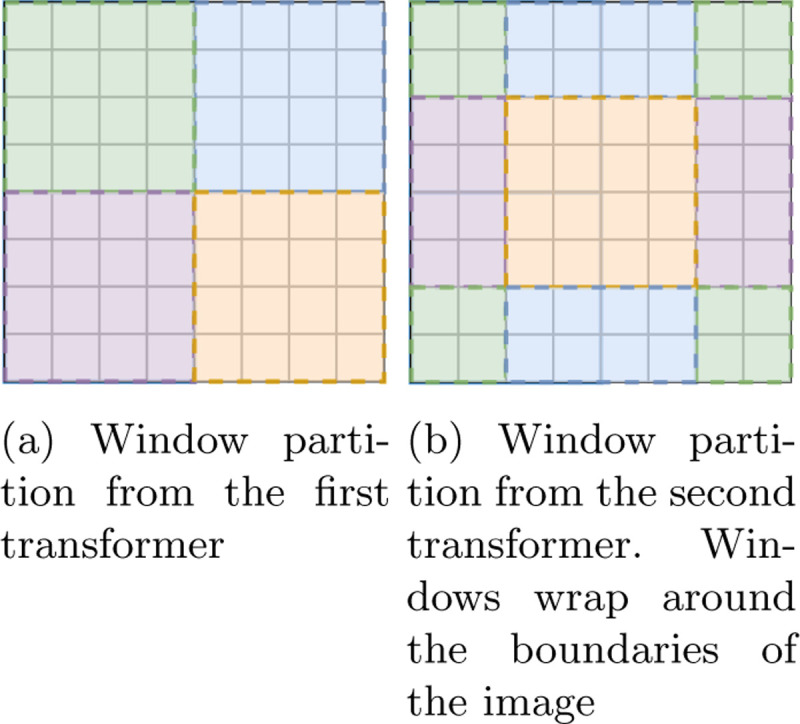
An example of shifted window partitioning for a Swin Transformer block. The window partition is cyclically shifted such that blind spots between the two are minimized. In this case, the partition is shifted two patches leftwards and two patches upwards.

Because time and space complexity of MSA grow exponentially with the number of patches [9], the patch set is split into non-overlapping windows, and MSA is computed for each window. The default Swin-Unet uses a window size of 7; we choose to keep as close to this number as possible while conforming to the shape of our input by using a window size of 6. With this windowing method alone, there would be no attention context at the borders of the windows, since MSA has only been computed within each window. For this reason, Swin Transformers come in pairs- the second transformer’s windows are shifted such that they build connections between the windows of the previous layer, eliminating any “blind spots” (see Fig. 2).

#### Patch Merging and Expansion:

Patch merging blocks serve as the network’s downsampling operation, similar to pooling in CNNs. In a patch merging block, each group of 2×2 adjacent depth-*d* patches are stacked into a single patch of depth 4*d*, then linearly projected down to 2*d*. As in CNN pooling, the set of patches has been downsampled by a factor of 4, and the remaining patches have greater depth. This enables a hierarchical representation of learnable features.

Similarly, patch expansion is the upsampling procedure of Swin-Uet, similar to transposed convolution upsampling in a vanilla UNet. The operation returns the set of patches to the same feature dimension that they had prior to patch merging. The set of patches is passed to a linear projection, then rearranged such that the height and width of each patch is doubled whilst the depth is halved. Finally, each of these spatially expanded patches is partitioned into a 2×2 group of smaller patches, returning them to their pre-merging dimension and quantity.

As in a CNN-based UNet, shallow features from the encoder are passed along via skip connections, and concatenated to deep features processed through the decoder. As with a vanilla UNet, this allows learning of shallow features unaffected by downsampling in addition to deep semantic features passed through the entire network.

Following the choice to make the input size of our Swin-Unet 48×48 is a reduction in depth of the network. Because our initial image size is small, patch merging becomes impossible at the bottom of the full network, as there is no deeper representation possible. We remedy this by simply removing one Transformer and Patch Merging block from the encoder, and one Transformer and Patch Expanding block from the decoder. As a result, there is no place for the usual skip connection at the deepest point of the network, so our model contains only two. As a result, we have only 4,990,032 learnable parameters, about a quarter of the 20,074,092 learnable parameters in a standard Swin-Unet.

### 3.2 Incremental Learning

In this section we describe the proposed incremental learning framework. We begin by training our multi-channel Swin-UNet to segment a large, multi-class, multi-channel image. Specifically, we chose half of a section to train this model, and tested/deployed the learned model on the other half. In a typical use of the Swin-Unet, train and test images are shaped and resampled to fit the 224 × 224 input size, such that every train and test case processed is a “full image/frame” of what is being segmented. In our case, we consider a multi-channel brain slice that is too large and high-resolution to conform to this input size. Increasing the input size to the Swin-Unet increases computation cost exponentially [9], so the large image is partitioned into patches to form the training set. Segmentation must similarly be performed patch-by-patch.

Our proposed incremental learning framework is centered on the following observation: A “global” multi-class segmentation is bound to be sub-optimal relative to each region that is being segmented. On the other hand, if the segmentation model is focused on a smaller subset of the image (i.e., if the problem is defined as segmenting a specific anatomical region from its surrounding regions), the complexity of the underlying task reduces. With this in mind, our framework uses the global segmentation task as a *prior* for fine-tuning the segmentation of individual anatomical regions. Specifically, we pre-train the network on the global segmentation task using all the available labeled training data, and then fine-tune the model for each anatomical region in the test region using a few labels (such as could be provided by a domain expert).

Given a segmentation of a large, multi-class, multi-channel image from the Swin-Unet model, an under-performing class is selected, and it’s region is isolated from the rest of the image. We then re-frame the segmentation as a binary problem rather than multi-class, considering only the class of interest and the background of the sub-region. Using a GUI that we developed for this purpose, a human domain expert may now visually form a new small binary training set by choosing patches from inside and outside the class region, and labelling each patch as belonging either to the class of interest or to its background. Note that because of the global pre-training, very few “clicks” are needed to fine-tune the model to any anatomical region of interest. For our GUI, we choose to build a plugin with Napari, which is a multi-dimensional image viewer [39].

In essence, we form a new binary Swin-Unet model seeded from the learned weights of the multi-class Swin-Unet model. We change the output layer to predict 2 classes instead of several, and train (fine-tune) the model with the human-defined “clicks” (set of patches). This can then be repeated until all regions of interest have been incrementally fine-tuned. Training (fine-tuning) this binary model is much faster than training the multi-class model because the feature extraction part of the network has already been pre-trained on a very similar (global segmentation) task using a large quantity of labeled training data, and, (2) the incremental training patches (no more than 30 training patches and often requires fewer than 10) from the test image provided by the domain expert steer the deep network to optimize itself for segmenting the specific region of interest.

The speed and ease of this procedure lends itself well to an incremental approach. The segmentation from the first pass of the binary model often contains inaccuracies that are clear to the human eye. However, it is straightforward to add or remove a few patches to account for the model’s mistake, and resume training. We repeat this procedure, fine-tuning and editing the training set repeatedly, until the segmentation can no longer be improved in a clear way by adding or removing patches. In a practical scenario, a domain expert can utilize visual cues and other context to provide these minimal labels from the test-domain for this task.

The result of this approach is a set of binary fine-tuned models for every class in the dataset. We achieve better segmentation performance with these models than could have been hoped for with a multi-class model, and have done so with a minimal amount of labelling effort during testing/deployment. Furthermore, by using this method it is not necessary to spend time and resources seeking the best possible multi-class segmentation; only a model good enough to seed the binary networks is required.

### 3.3 Sliding-Window Inference Smoothing

Because the image we would like to perform segmentation on is larger than can be reasonably fit into the input of a Swin-Unet model, the image must be split into patches, which are segmented individually. A consequence of this approach is distortion of the segmentation at edges where the patches meet. This can result in a “blocky” or “pixelated” segmentation that hurts performance.

To remedy this, we propose a sliding-window inference method. Our goal is to eliminate artifacts where patches meet by forming additional, overlapping patch sets at different 2D offsets, computing inference (segmentation prediction) over the patches at each of these offsets, then combining the set of overlapping offset inference windows in a way that will smooth the segmentation.

We choose some integer f which is a factor of the image size, 48. We define the number of unique inference windows as $n={\left(\frac{48}{f}\right)}^{2}$, and compose each of the offset values x and y from the set:

(2) $$x,y\in \{\frac{m\times f}{48},m\in 0,1,\ldots ,n\}.$$

Finally, we form the output inference map $W^{\mathrm{smoothed}}$ by taking the statistical mode of each predicted pixel from the offset windows. That is,

(3) $$W_{i,j}^{smoothed}=\underset{x,y}{\text{mode}}\left\{W_{i,j}^{x,y}\right\},$$

where $W^{x,y}$ is the inference window at offset x,y, and it’s pixels are indexed by i,j. Smaller values of f give a greater number of windows and therefore a smoother inference, at the expense of more computational resources. Note that setting f to 48 (the patch size) is equivalent to standard (non-windowed) inference. In this work, we choose the smallest possible value, f=4, that can be accommodated with our computational resources, given the large size of the image.

## 4 Experimental Setup and Results

### 4.1 Dataset Description

In this work, we use imagery of a rat brain section, acquired using our large-scale highly multiplexed immunoflourescence imaging framework [24]. The original imagery comprises of 50 high-resolution (29398 × 43054 pixels) biomarker channels scanned from a single rat brain slice, with each biomarker identifying a specific resident cell type and its unique cellular distributions in different anatomically-identifiable parts of the brain, which match the classical atlas regions previously mapped using traditional using low-plex Nissl and Hematoxylin-Eosin brigfield imaging techniques [40]. In this work, we downsampled the slice by a factor of 5 to 5880 × 8622 pixels to fit our computational environment (GPU memory). We identify 10 anatomical regions of interest which we seek to accurately segment:

- Corpus callosum (*cc*)
- Medial habenular nucleus (*MHb*)
- Reticular thalamic nucleus (*Rt*)
- Mammillothalamic tract (*mt*)
- Stigmoid hypothalamic nucleus (*Stg*)
- Medial globus pallidus (*MGP*)
- Ventromedial hypothalamic nucleus (*VMH*)
- Optic tract (*opt*)
- Fimbria of the hippocampus (*fi*)
- Stria medullaris of the thalamus (*sm*)

7 biomarkers for which the regions of interest are most visible are selected to form a 7-channel image. We chose the following 7 biomarkers to form our channels:

- CNPase (oligodentrocytes, soma and processes-specific)
- GFAP (astrocytes, processes-specific)
- NeuN (neurons, nucleus and soma-specific)
- OLIG2 (oligodendrocytes, nucleus-specific)
- Parvalbumin (interneurons, soma and processes-specific)
- S100 (astrocytes, nucleus and soma-specific)
- Tyrosine Hydroxylase (catecholaminergic neurons, soma and processes-specific)

**Fig. 3:**
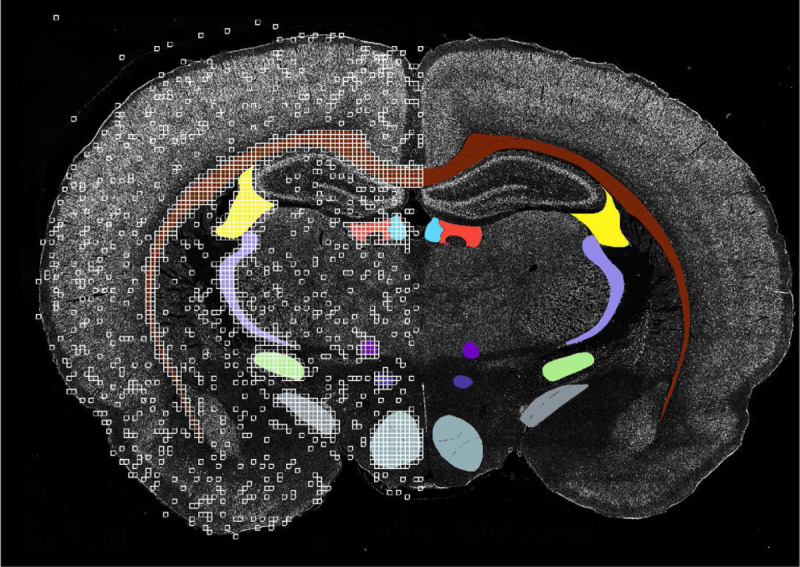
One channel of the brain image overlaid with the ground truth labels for all 10 classes. The patches forming the training set are shown as white squares.

Because the brain sections are roughly symmetrical, the training data for the Swin-Unet model is formed from selected patches on the left half of the brain image, while the right half is reserved for testing the model’s performance. To form the training set, the image is partitioned into 8 × 48 × 48 slices. Any slice in the left half of the image that contains one of the 10 regions of interest is added to the training set, plus 10% of all other slices, randomly sampled (See Fig.3). A threshold is applied to prevent training on the dark background of the brain image. We test the model on the right half of the section using the sliding-window inference described previously. We use the Intersection over Union (IoU) metric to quantify and compare performance of the resulting segmentations. IoU is defined:

(4) $$IoU=\frac{A\cap B}{A\cup B}$$

Where A is the model segmentation and B is the ground truth annotation.

### 4.2 Experimental Setup and Results

**Fig. 5:**
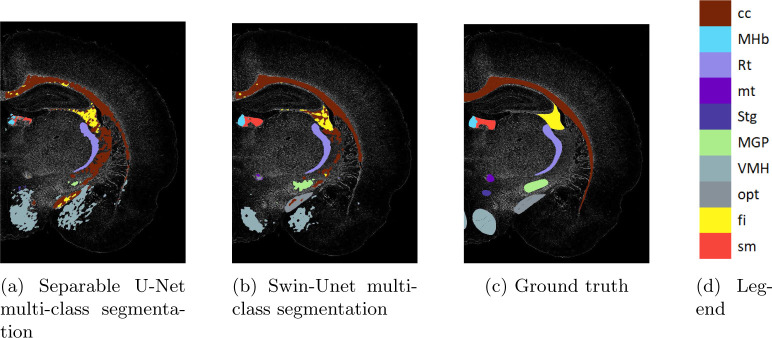
Performance of multi-class models.

We first show the multi-class inference results of our custom Swin-Unet on the dataset, and ablate with a comparable Unet model (see Fig.5). We use an off-the-shelf UNet for this comparison with a single modification: the convolutions in the UNet model have been made separable for a better comparison with our modified Swin-Unet model on multi-channel data. Table 1 shows the IoU scores for each region and the average IoU across all regions, for each model. The custom Swin-Unet performs better than the separable UNet by a large margin on most classes.

Using our multi-channel Swin-Unet on the multiplex IF microscopy imagery produces the segmentation shown in Fig.5. Some regions are well-segmented, while others are poorly segmented (e.g. over-segmented and under-segmented). One of the classes was not predicted at all. Fig.4 shows the results of deploying two shots of the proposed incremental learning framework for each of the 10 regions of interest from the original segmentation, and Table 2 shows the IoU for each of these segmentations.

When applying the multi-class model to each individual region, we frame the problem as a binary one rather than multi-class, i.e., any segmentation made for a region besides the region of interest is treated as a common background class. This will allow for a straightforward comparison between the multi-class model and the fine-tuned binary models.

The fine-tuned models outperformed the multi-class model in every region but two, which lagged by .02 and .03 IoU points. In the case of the Rt region, the multi-class segmentation was so exceptional (0.97 IoU) that there was not much improvement to be gained by applying our method in the first place. To show global performance, we consider the IoU of every region and take the mean. The mean IoU of the multi-class model was 0.62, while the mean of the second and final iteration of the fine-tuned binary models was 0.81, a global IoU improvement of 0.19.

For analysis of the performance of individual regions, it is useful to consider these regions in terms of the following three types:

1. Regions that have textures and edges plainly visible to the human eye in at least one channel (biomarker). These regions can be easily segmented by an untrained human.
2. Regions that have few or no human-visible edges, but have a texture or cellular structure that differs from the surrounding regions. These regions may have enough context between all channels of the image for a model to make a good segmentation, but would be difficult for a human to precisely discern.
3. Regions that are not visible to the human eye, and cannot be distinguished from surrounding regions. These regions are practically impossible for a human to segment through visual cues.

Type 1 regions include *MHb*, *Rt*, and *sm*. These regions were segmented well by the multi-class model, so local fine-tuning yielded little or no improvement. Type 2 regions include *MGP*, *VMH*, *opt*, and *fi*. All of these regions saw major improvement from incremental learning. Type 3 regions include *mt* and *Stg*. Both of these very small regions saw large improvements from incremental learning. *cc* is a particular region of interest, because it’s inference region practically spans the entire image. One might expect a large amount of false-positive segmentations due to having so many different regions in the same domain, but the fine-tuned model is able to identify the region of interest reasonably well in two rounds with plenty of labelling. Additionally, the fine-tuned model captures the lower part of the *cc* region, which the multi-class model was unable to segment. This demonstrates the scalability of this approach to large regions.

The multi-class model takes about 30 minutes to train for 1000 epochs, whereas fine-tuning each region takes 1 to 4 minutes, requiring at most 400 epochs, but more typically around 100 epochs. All training is done on a cluster of 3 Nvidia GeForce GTX Titan X GPUs. Inference time varies by region. Inference for the full multi-class image and the large cc region both take about 25 minutes on the above hardware. For the remaining regions, inference time is under 4 minutes.

**Fig. 6:**
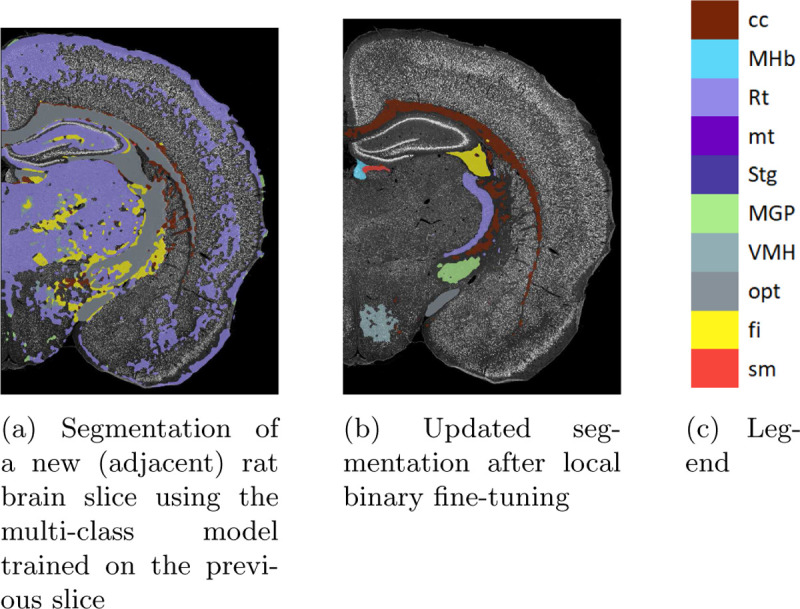
Segmentation of a new rat brain slice before and after local fine-tuning. The fine-tuning result is achieved with an average of about 25 user-provided mouse clicks per region, excluding the large cc region (111 clicks).

As a supplemental demonstration of the efficacy of our incremental fine-tuning method, we apply the multi-class model, without any additional fine-tuning, to an adjacent brain slice. Predictably, the initial segmentation is poor, as the variability between sections can negatively impact segmentation performance. There are large swaths of mis-segmented background regions, and, though there are many regions that are localized correctly, they are labelled incorrectly. Once again, local fine-tuning of the multi-class model provides better segmentations. Although there is no ground truth atlas for this brain slice, the improvement is visually apparent in Fig.6

## 5 Conclusion

In this work, we introduced an incremental learning framework that leverages the representation power of a multi-channel Swin-UNet for semantic segmentation of multi-channel IF microscopy images. We validated this approach on a multiplex IF image representing a rat brain section. Our model improved the segmentation of 8 out of 10 regions, and improved the average overall IoU by 0.19. Further, starting from an appropriate pre-training, our work shows that this framework can be used to incrementally (and efficiently) adapt the model to different images and different regions within an image (e.g. different subjects, different sections) with minimal user input in terms of additional labeling. This framework can also be readily utilized for other biomedical semantic segmentation tasks as well.

## Figures and Tables

**Fig. 4:**
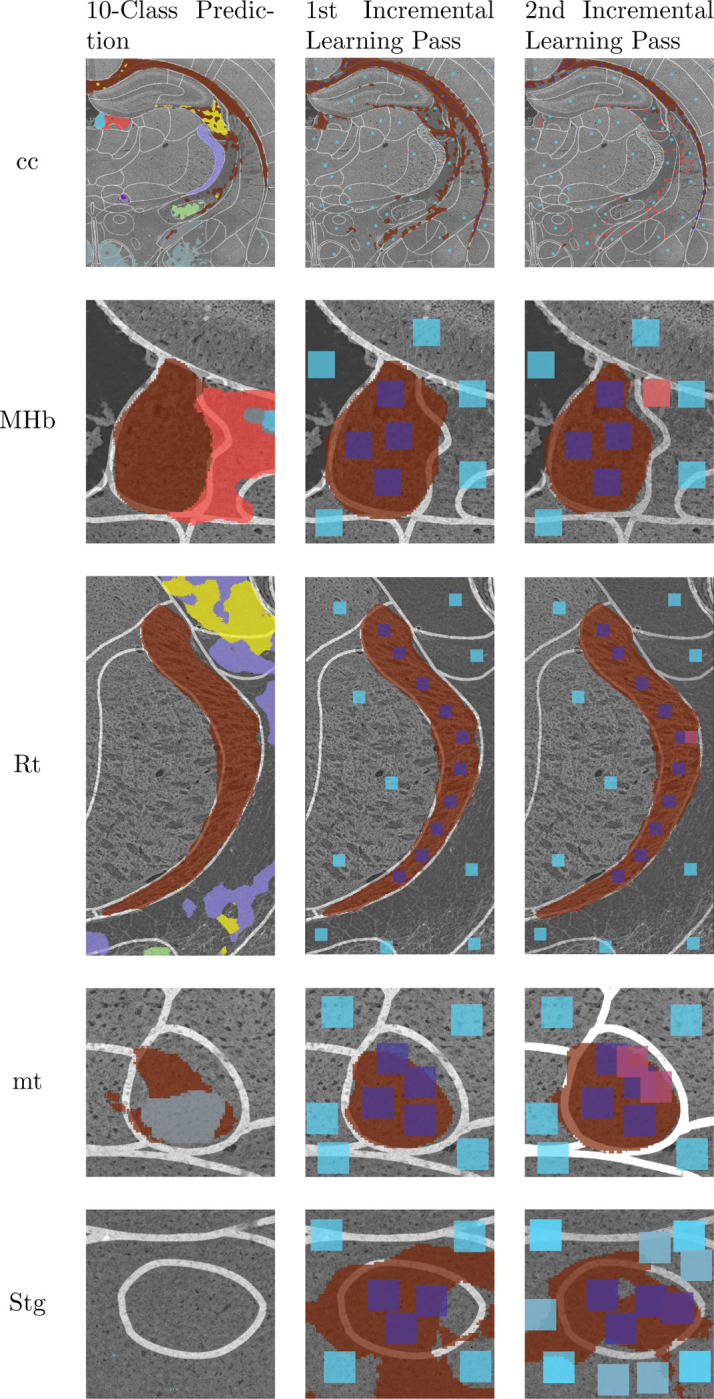
Improving segmentation results with human-in-the-loop incremental learning.

**Fig. 4:**
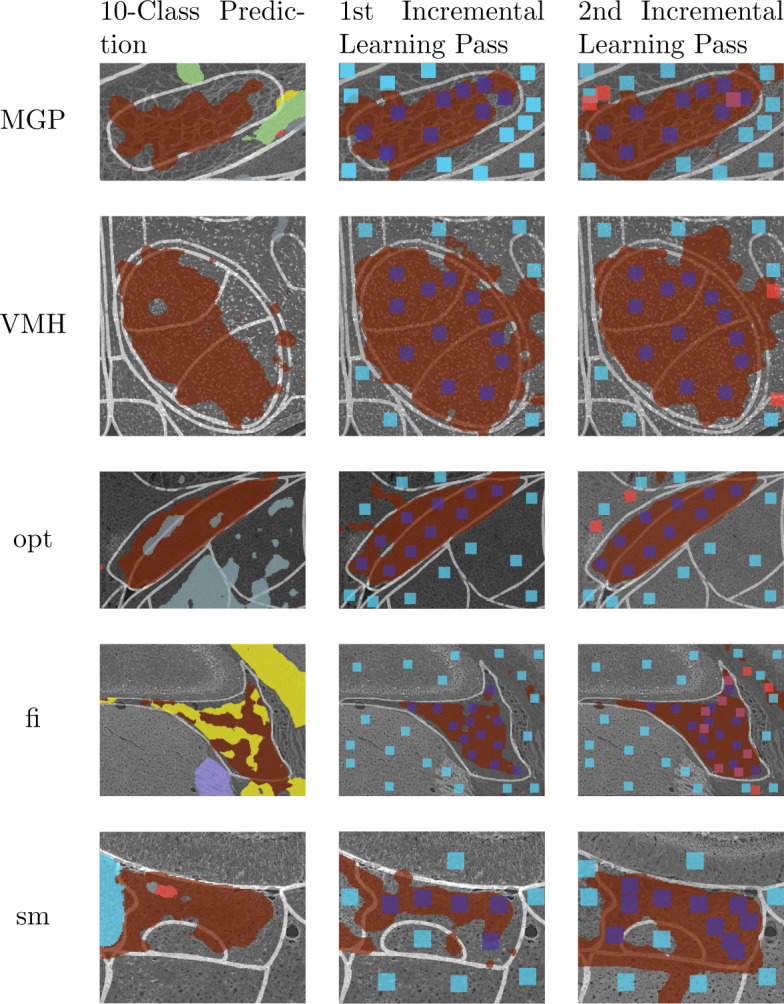
Improving segmentation results with human-in-the-loop incremental learning. The “clicks” are indicated by patches. Clicks in the second pass of incremental learning are indicated by red colored patches.

**Table 1:** Multi-class IOU scores for the separable UNet and full-parameter Swin-Unet

Region	UNet IoU	Swin-Unet IoU

cc	0.43	0.59
MHb	0.48	0.97
Rt	0.84	0.94
mt	0.01	0.25
Stg	0.05	**DNP** ^1^
MGP	0.12	0.66
VMH	0.30	0.72
opt	0.00	0.79
fi	0.49	0.58
sm	0.26	0.69

Average	0.30	0.62

1 Did not predict

**Table 2:** Improving segmentation results with human-aided binary fine-tuning of local regions. The segmentations in the left column are multi-class; segmentations of the class of interest are colored brown. The other two columns show the patches used to fine-tune the binary model that generated the shown segmentation.

Region	Multi-class Prediction IoU	1st Binary Fine-Tune IoU	2nd Binary Fine-Tune IoU

cc	0.59	0.41	0.70
MHb	0.97	0.94	0.95
Rt	0.94	0.97	0.96
mt	0.25	0.81	0.91
Stg	DNP^1^	0.41	0.68
MGP	0.66	0.78	0.82
VMH	0.72	0.79	0.78
opt	0.79	0.85	0.89
fi	0.58	0.60	0.77
sm	0.69	0.54	0.64

Average	0.62	0.79	0.81

1 Did not predict
